# Genetic defect of the sodium‐dependent multivitamin transporter: A treatable disease, mimicking biotinidase deficiency

**DOI:** 10.1002/jmd2.12040

**Published:** 2019-05-28

**Authors:** Marit Schwantje, Monique de Sain‐van der Velden, Judith Jans, Koen van Gassen, Charlotte Dorrepaal, Klaas Koop, Gepke Visser

**Affiliations:** ^1^ Department of Genetics University Medical Center Utrecht Utrecht The Netherlands; ^2^ Department of Metabolic Diseases Wilhelmina Children's Hospital, University Medical Center Utrecht Utrecht the Netherlands; ^3^ Department of Pediatrics St. Antonius Hospital Woerden The Netherlands

## Abstract

The sodium‐dependent multivitamin transporter that facilitates the uptake of the water‐soluble vitamins biotin, pantothenic acid, and the vitamin‐like substance lipoate is coded by the *SLC5A6* gene. Variants in this gene cause a relatively novel treatable metabolic disorder. Here we describe the second case. A 17‐month‐old girl presented with hypoglycemia (2.0 mmol/L) and severe metabolic acidosis (pH 6.87), leading to resuscitation. Her history revealed feeding problems from birth and poor weight gain. Metabolic investigation showed elevated plasma C3‐carnitine and C5‐OH‐carnitine. Urine analysis showed persistently elevated excretion of 3‐OH‐isovaleric acid. Biochemically, the combination of elevated C5‐OH‐carnitine and increased excretion of 3‐OH‐isovaleric acid seemed compatible with biotinidase deficiency. Supplementation with biotin was started. Biotinidase activity in plasma showed only marginally decreased activity, which was considered insufficient explanation for her clinical symptoms. Subsequent trio‐based whole exome sequencing revealed compound heterozygosity for variants in the *SLC5A6* gene. Upon increasing the dosage of biotin supplementation and introduction of pantothenic acid supplementation, a striking clinical improvement was seen.

**Synopsis**

Variants in the *SLC5A6* gene may lead to a metabolic disorder mimicking biotinidase deficiency that can be treated with supplementation of the vitamins biotin and pantothenic acid.

## 1 INTRODUCTION

The uptake of the water‐soluble vitamins biotin, pantothenic acid, and the vitamin‐like substance lipoate is facilitated by the sodium‐dependent multivitamin transporter (SMVT). All vitamins play a crucial role in energy metabolism of the human body. The SMVT has been detected in multiple tissue types, including liver, kidney, and placenta tissues.^1^, ^2^ SMVT is the only biotin transport system that operates in the intestine, and its knockout leads to biotin deficiency.^3^ Biotin (vitamin B7) serves as an activating cofactor of the five carboxylases involved in a variety of metabolic reactions, including fatty acid synthesis, gluconeogenesis, and amino acid catabolism.^4^ Pantothenic acid is the precursor of coenzyme A and therefore pantothenic acid deficiency can lead to a limited availability of coenzyme A. Coenzyme A operates as a cofactor of enzymes involved in the tricarboxylic acid cycle and fatty acid metabolism.^5^, ^6^ Lipoate is one of the cofactors in the glycine cleavage system and the pyruvate dehydrogenase, branched chain keto acid dehydrogenase, and ketoglutarate dehydrogenase complexes. These enzymes catalyze redox reactions in the mitochondrial energy production and enable oxidative decarboxylation reactions of amino acids and keto acids.^7^, ^8^, ^9^, ^10^, ^11^ Next to its function as cofactor, lipoate might also have anti‐oxidative and anti‐inflammatory effects.^12^ In addition to facilitating the uptake of biotin, pantothenic acid, and lipoate, SMVT is able to transport iodide and therefore possibly also influences the iodide homeostasis of the body. However, its exact role in iodide transport and homeostasis is still unknown.^13^

The gene coding for SMVT, *SLC5A6*, is located on chromosome 2.^2^ Variants leading to an impaired SMVT protein function might result in an intracellular deficiency of its organic substrates, as it is the only known combined biotin, pantothenic acid, and lipoate uptake transporter.^1^, ^14^ Considering their metabolic functions, intracellular deficiencies of biotin, pantothenic acid, and lipoate can cause a wide variety of signs and symptoms. The recent report of an infant with variants in the *SLC5A6* gene with severe clinical findings supports this hypothesis.^15^ At 15 months of age, this infant presented with severe feeding problems, failure to thrive, microcephaly, and generalized atrophy of the brain on MRI. He had a cerebral palsy and a developmental delay, a variable immunodeficiency, and a severe gastroesophageal reflux. Also, pathologic bone fractures caused by osteoporosis occurred. Supplementation of biotin, pantothenic acid, and lipoate led improvement of his condition.

Here, we describe a second case with variants in the *SLC5A6* gene, who presented with signs and symptoms mimicking biotinidase deficiency. We discuss the similarities and differences in clinical presentation and outcome between these two patients.

## 2 CASE REPORT

The patient, a now 3‐year‐old girl, is the fifth child of healthy, non‐consanguineous parents. She was born full‐term, after an uncomplicated pregnancy. Apgar scores were 10 and 10 after 1 and 5 minutes. Her birth weight was 3.1 kg and she had no dysmorphic features. Because of her parents' religious convictions, she was not vaccinated and is home schooled.

Growth and development were normal during the first few months of her life. At the age of 10 months, she was admitted to a local hospital because of vomiting with traces of blood. Antacid treatment was started for a suspected Mallory Weiss syndrome. In contrast to her older siblings, she still was fully breastfed at that age as she rejected introduction of solid foods. Despite logopedic therapy, feeding problems persisted and she showed poor weight gain (−2 SD). Her speech development progressed normal, but she had a delay in gross motor development.

At 17 months of age, she was readmitted with reduced consciousness and circulatory insufficiency during gastroenteritis. She had a severe metabolic acidosis: pH 6.87 (normal range: 7.37‐7.45), base excess −30 mmol/L (normal range: −3.0 to 3.0 mmol/L), pCO_2_ 24.6 mm Hg, hypoglycemia 2.0 mmol/L (normal range: 3.6‐5.6 mmol/L), hyperammonemia 200 μmol/L (normal range: 0‐35 μmol/L), a mildly elevated lactate 3.0 mmol/L (normal range: 0‐2.2 mmol/L), and ketonuria.

She needed respiratory and circulatory support, and was intubated. During intubation, she developed ventricular fibrillation requiring cardiopulmonary resuscitation. She was transferred to our center and was weaned from ventilator support after correction of her acidosis, and replacement of fluid and electrolyte deficits. Metabolic investigation at the age of 1 year showed elevated plasma C3‐carnitine (2.20‐1.88‐7.4 μmol/L; upper limit: 0.81 μmoL/L) and C5‐OH‐carnitine (0.16‐0.17‐0.23 μmol/L; upper limit: 0.02 μmol/L). Urine analysis showed persisted elevated excretion of 3‐OH‐isovaleric acid (2193‐504‐146 mmol/mol creatinine; upper limit: 67 mmol/mol creatinine).

The combination of elevated C5‐OH‐carnitine and increased excretion of 3‐OH‐isovaleric acid may suggest a metabolic disorder (eg, beta ketothiolase deficiency, multiple carboxylase deficiency, and biotinidase deficiency). Biotinidase activity analysis in plasma showed a marginal decreased enzyme activity (4.2 nmol PABA/mL/min; normal range: 4.6‐11.6 nmol PABA/mL/min). This was considered insufficient to explain her clinical symptoms. However, the biochemical and clinical similarities with a biotinidase deficiency suggested an intracellular biotin deficiency. For that reason, biotin supplementation (5 mg twice a day) was initiated. She made a good clinical recovery. Upon evaluation, apart from possible mild ischemic changes, no abnormalities were seen on MRI. She was discharged with a nasogastric tube to provide optimal feeding. A fasting test performed at the age of 20 months showed a normal fasting tolerance and the tube feeding was terminated at the age of 24 months.

Because of the severity of her metabolic derangement and ongoing distress with feeding problems, the parents agreed further diagnostic evaluation by trio‐based whole exome sequencing (trio‐WES). This revealed the compound heterozygous variants NM_021095.2: c.422_423del p.(Val141fs) and c.1865_1866del p.(Gln622fs) in the SLC5A6 gene. This gene codes for the SMVT. Parents were each heterozygous for one of the variants.

The genetic diagnosis was established at the age of 3 years. Before diagnosis, she had been treated with biotin supplements (5 mg twice a day). Her delay in gross motor development remained stable (−2 SD). Feeding problems still existed, resulting in poor weight gain (−1.6 SD) and a deviating height (−2.4 SD). Therefore, introduction of medical drink feeding was necessary. She had chronic diarrhea and sporadic vomiting. After diagnosis, the biotin dose was doubled to twice a day 10 mg and pantothenic acid (250 mg/day once a day) was added. Since then, her appetite increased, diarrhea resolved, and growth improved. But she still showed a delayed gross motor development of −2 SD (Bailey Scales of Infant Development‐III).

Upon follow‐up, biotinidase activity had normalized (11 nmol PABA/mL/min; normal range: 4.6‐11.6 nmol PABA/mL/min) and plasma amino acids were within the normal range. Also, her biotinidase newborn screening result was normal.

Thyroid‐stimulating hormone (TSH) was slightly increased 7.3 (0.35‐5.0 mU/L), while free T4 16 (11‐20 pmol/L), total T4 103 (80‐180 nmol/L), and T3 2.4 (1.0‐3.0 nmol/L) were normal. Her newborn brother was tested for variants in the *SLC5A6* gene; he turned out to be carrier of the c.1865_1866del p.(Gln622fs) variant.

## 3 DISCUSSION

This case report illustrates the possible severe consequences of pathogenic variants in the *SLC5A6* gene and the clinical resemblance to biotinidase deficiency. In conditional *SLC5A6* KO mice, biotin and pantothenic acid supplementation prevent the development of intestinal mucosal abnormalities and growth defects.^16^ The present case and the patient of Subramanian et al^15^ both showed a remarkable positive clinical response upon supplementation. SMVT deficiency is therefore a potentially treatable metabolic disorder. Although WES was necessary to diagnose our patient, metabolic abnormalities in urine and plasma pinpointed the involvement of biotin metabolism.

The patient presented with severe metabolic acidosis and hypoglycaemia and was hyperpnoeic, as evidenced by her low pCO_2_ during an infection with fever. These biochemical abnormalities were not seen previously, nor were they seen in the initial patient, illustrating the fact that infections may unmask this disorder and metabolic sample taking during infections might pinpoint to the diagnosis. The case furthermore shows that intubation in a setting of severe metabolic acidosis can be a very dangerous procedure. Anesthetic medication given during intubation will limit the patient's respiratory effort. Unless immediate ventilation is started, this will lead to an increase in pCO_2_, further decrease of pH, and may eventually lead to cardiac arrest.

In retrospect, the hypoglycemia may have been caused by an intracellular pantothenic acid deficiency, as this leads to an insufficient availability of coenzyme A and a following impaired fatty acid metabolism.^5^ Hypoglycaemia due to pantothenic acid deficiency has been previously reported in animal models and humans.^5^

During follow‐up, she developed chronic diarrhea. An impaired function of the SMVT and in particular an intracellular biotin deficiency negatively influences the maintenance of the intestinal mucosa integrity.^17^ This possibly caused the chronic diarrhea, since it disappeared upon doubling the biotin dose. However, supplementation of pantothenic acid was at that time also initiated, hence this may also have contributed to the marked clinical improvement.

Lipoate supplementation was not initiated upon the discovery of variants in the *SLC5A6* gene, because a deficiency was considered unlikely given the normal concentrations of glycine and pyruvate. This might be explained by the possibility of de novo synthesis of lipoate in humans.^12^, ^18^

In addition to the described metabolites, a role of SMVT in the cellular iodine transport has been identified. However, the exact relevance in iodine homeostasis is still unclear.^13^ Theoretically, an impaired SMVT function can lead to a reduced thyroid function with a low T4 level and high TSH. Testing thyroid function in this patient showed normal T4 and T3 levels but TSH was slightly elevated and will be monitored.

A remarkable resemblance between the two reported cases with variants in the *SLC5A6* gene was the presence of severe and early onset feeding problems. Feeding problems were so severe that supplementary feeding and even gastrostomy or tube feeding had to be introduced in both. However, the microcephaly, immunologic disorders, cerebral palsy, and fractures due to osteoporosis in the index patient, were absent in our patient. In addition, the index patient had a severe development delay, while our patient only had slight gross motor development delay. The relatively mild clinical presentation in our patient could be explained by residual functioning of the c.1865_1866del p.(Gln622fs) variant in the *SLC5A6* gene. This variant causes a frameshift in the last exon of the *SLC5A6* gene, that, if the protein remains stable, will extend the protein by 37 amino acids.

Although the full clinical spectrum of a defect in SMVT remains to be delineated, both patients responded positively to supplementation of biotin and pantothenic acid. Apparently, when provided in high doses, intracellular concentrations of these SMVT substrates become sufficient for enzyme functioning, leading to a clinical improvement.

In conclusion, recessive variants in the *SLC5A6* can cause a clinically relevant SMVT defect, causing a treatable metabolic disorder that mimics biotinidase deficiency with characteristic feeding problems and varying symptoms, caused by intracellular deficiencies of biotin, pantothenic acid, and lipoate.

## AUTHOR CONTRIBUTIONS

M.S., G.V., K.K., and C.D. were involved in diagnostic and treatment of the patient, and the collection of clinical data; K.L.I.G., J.J., and M.G.M.S.V. were involved in metabolic analyses and interpretation of the patient's data. M.S., G.V., and M.G.M.S.V. were involved in the writing and editing of the manuscript. All authors have given final approval of the version to be published.

## CONFLICT OF INTEREST

The authors declare no potential conflicts of interest.
